# Reversal of sorafenib resistance in hepatocellular carcinoma: epigenetically regulated disruption of 14-3-3η/hypoxia-inducible factor-1α

**DOI:** 10.1038/s41420-019-0200-8

**Published:** 2019-07-19

**Authors:** Yongxin Qiu, Wenqi Shan, Ye Yang, Ming Jin, Yi Dai, Hanyu Yang, Ruonan Jiao, Yunwei Xia, Qinqiang Liu, Liang Ju, Guangming Huang, Jianping Zhang, Lihua Yang, Lei Li, Yuan Li

**Affiliations:** 1 Jiangsu Key Lab of Cancer Biomarkers, Prevention and Treatment, Collaborative Innovation Center for Cancer Personalized Medicine, School of Public Health, Nanjing Medical University, Nanjing, 211166 China; 2 Department of Medical Center for Digestive Diseases, The Second Affiliated Hospital, Nanjing Medical University, Nanjing, 210011 China; 3 Key Laboratory of Modern Toxicology, Ministry of Education, School of Public Health, Nanjing Medical University, Nanjing, 211166 China

**Keywords:** Oncogenes, Cell signalling

## Abstract

Sorafenib resistance is one of the main obstacles to the treatment of advanced/recurrent hepatocellular carcinoma (HCC). Here, sorafenib-resistant HCC cells and xenografts in nude mice were used as experimental models. A cohort of patients with advanced recurrent HCC who were receiving sorafenib therapy was used to assess the clinical significance of this therapy. Our data showed that 14-3-3η maintained sorafenib resistance in HCC. An analysis of the underlying molecular mechanisms revealed that 14-3-3η stabilizes hypoxia-inducible factor 1α (HIF-1α) through the inhibition of ubiquitin-dependent proteasome protein degradation, which leads to the maintenance of cancer stem cell (CSC) properties. We further found that microRNA-16 (miR-16) is a competent miRNA that reverses sorafenib resistance by targeting the 3′-UTR of 14-3-3η and thereby inhibits 14-3-3η/HIF-1α/CSC properties. In HCC patients, significant negative correlations were found between the expression of miR-16 and 14-3-3η, HIF-1α, or CSC properties. Further analysis showed that low miR-16 expression but high 14-3-3η expression can prognosticate sorafenib resistance and poor survival. Collectively, our present study indicated that miR-16/14-3-3η is involved in sorafenib resistance in HCC and that these two factors could be potential therapeutic targets and biomarkers for predicting the response to sorafenib treatment.

## Introduction

Hepatocellular carcinoma (HCC) is one of the most common solid tumors and the leading cause of cancer-related mortality worldwide^1,2^. Because most patients with HCC lose the opportunity for radical treatment (operation or liver transplantation) due to the advanced stage at which the cancer is detected, the long-term outcome of HCC is poor^3^. To date, sorafenib is the only approved systemic therapy for the treatment of advanced and recurrent HCC^4^, but its therapeutic effect is less than satisfactory, largely due to hypoxia-mediated sorafenib resistance^5–7^. Indeed, hypoxia induced by sustained sorafenib treatment confers resistance through the activation of hypoxia-inducible factor 1α (HIF-1α), which leads to the generation of cancer stem-like cells (CSCs)^8–10^. Consequently, the continued search for novel therapeutic strategies targeting HIF-1α-regulated CSC properties in HCC is urgently needed.

The 14-3-3 proteins are a family of approximately 28–33-kDa acidic polypeptides that regulate multiple cellular functions via interactions with intracellular proteins^11–15^. Our previous study identified 14-3-3η as a novel angiogenic factor in HCC, and this factor can also be considered a biomarker for predicting the response to sorafenib treatment^16^. Using computer docking software (PyMOL), we found that 14-3-3η can bind to HIF-1α, which suggests an interaction between these two proteins. However, the functions of 14-3-3η in HIF-1α/CSC-mediated sorafenib resistance remain largely uninvestigated. Moreover, despite the well-defined outcome of 14-3-3η overexpression in HCC, the mechanism underlying its upregulation remains unclear.

MicroRNAs (miRNAs) are a group of small noncoding RNAs that negatively regulate the expression of their target genes at posttranscriptional levels by directly binding to the 3′-untranslated regions (UTRs) of these genes^17–19^. Through a high-throughput miRNA microarray analysis, our previous study revealed that 28 miRNAs were significantly hypo-expressed in the poorly differentiated group (relatively enhanced CSC properties) compared with their expression in well-differentiated HCC tissues (relatively suppressed CSC properties)^20,21^. Through a further combined analysis using a web-based miRNA resource (TargetScan 7.1), we identified a candidate miRNA, miR-16, that might regulate 14-3-3η expression. Previous studies have demonstrated that miR-16 is an important tumor suppressor in HCC^22–24^ and that a lack of miR-16 might render tumors resistant to chemotherapy drugs such as fluorouracil and cisplatin^25,26^. Therefore, our present study aimed to investigate the relationships between miR-16 and 14-3-3η and their roles in HIF-1α-induced CSC properties and sorafenib resistance.

## Results

### 14-3-3η induced/maintained CSC properties and sorafenib resistance

**Fig. 1:**
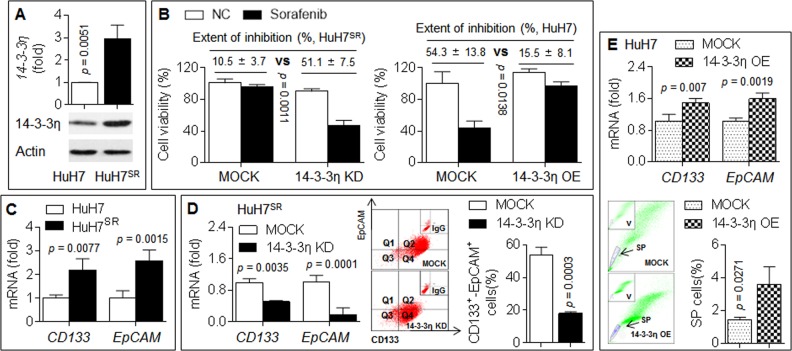
14-3-3η induced/maintained CSCs properties and sorafenib resistance. **a** qPCR in triplicate and IB analysis of the expressions of 14-3-3η mRNA (top) and protein (bottom) in HuH7 and HuH7^SR^ cells. **b** HuH7^SR^ cells were transfected by scrambled or 14-3-3η siRNA (14-3-3η KD), while HuH7 cells were transfected by scrambled or 14-3-3η plasmid (14-3-3η OE), after then, they were treated with sorafenib. Cell viabilities were analyzed in triplicate by CCK-8 solution. **c** qPCR analysis in triplicate of the expressions of *CD133* and *EpCAM* mRNAs in HuH7 and HuH7^SR^ cells. **d** HuH7^SR^ cells were transfected by scrambled or 14-3-3η siRNA, qRT-PCR analysis of the expressions of *CD133* and *EpCAM* mRNAs (left); flow cytometry analysis in triplicate of the ratio of CD133^+^-EpCAM^+^ cells (right). **e** HuH7 cells were transfected by scrambled or 14-3-3η plasmid, qRT-PCR analysis of the expressions of *CD133* and *EpCAM* mRNAs (top); flow cytometry analysis in triplicate of the ratio of SP cells (bottom)

First, we produced sorafenib-resistant HuH7 cells (HuH7^SR^) and found that the expression of 14-3-3η was increased in HuH7^SR^ cells compared with their parental counterparts (Fig. 1a). We then knocked down 14-3-3η using its specific siRNA. Compared with the scramble group, the 14-3-3η siRNA-transfected cells showed a recovered response to sorafenib, as determined by a decreased cell viability; in contrast, the overexpression of 14-3-3η in HuH7 cells exerted the opposite effects (Fig. 1b).

Previous studies revealed that long-term sorafenib treatment resulted in an enhancement of CSC properties and thereby induced sorafenib resistance in HCC cells^4,10^. In the present study, the expression of CD133 and EpCAM in HuH7^SR^ was significantly higher than that in parental HuH7 cells (Fig. 1c). The knockdown of 14-3-3η in HuH7^SR^ cells attenuated the expression of *CD133* and *EpCAM* and decreased the ratios of CD133^+^–EpCAM^+^ cells (a biomarker for CSC properties, Fig. 1d). In contrast, the overexpression of 14-3-3η in HuH7 cells enhanced the expression of *CD133* and *EpCAM* and increased the ratios of SP cells (another biomarker for CSC properties, Fig. 1e). Collectively, these results suggested that 14-3-3η induced/maintained CSC properties and thereby induced sorafenib resistance in HCC cells.

### 14-3-3η stabilized and activated HIF-1α

**Fig. 2:**
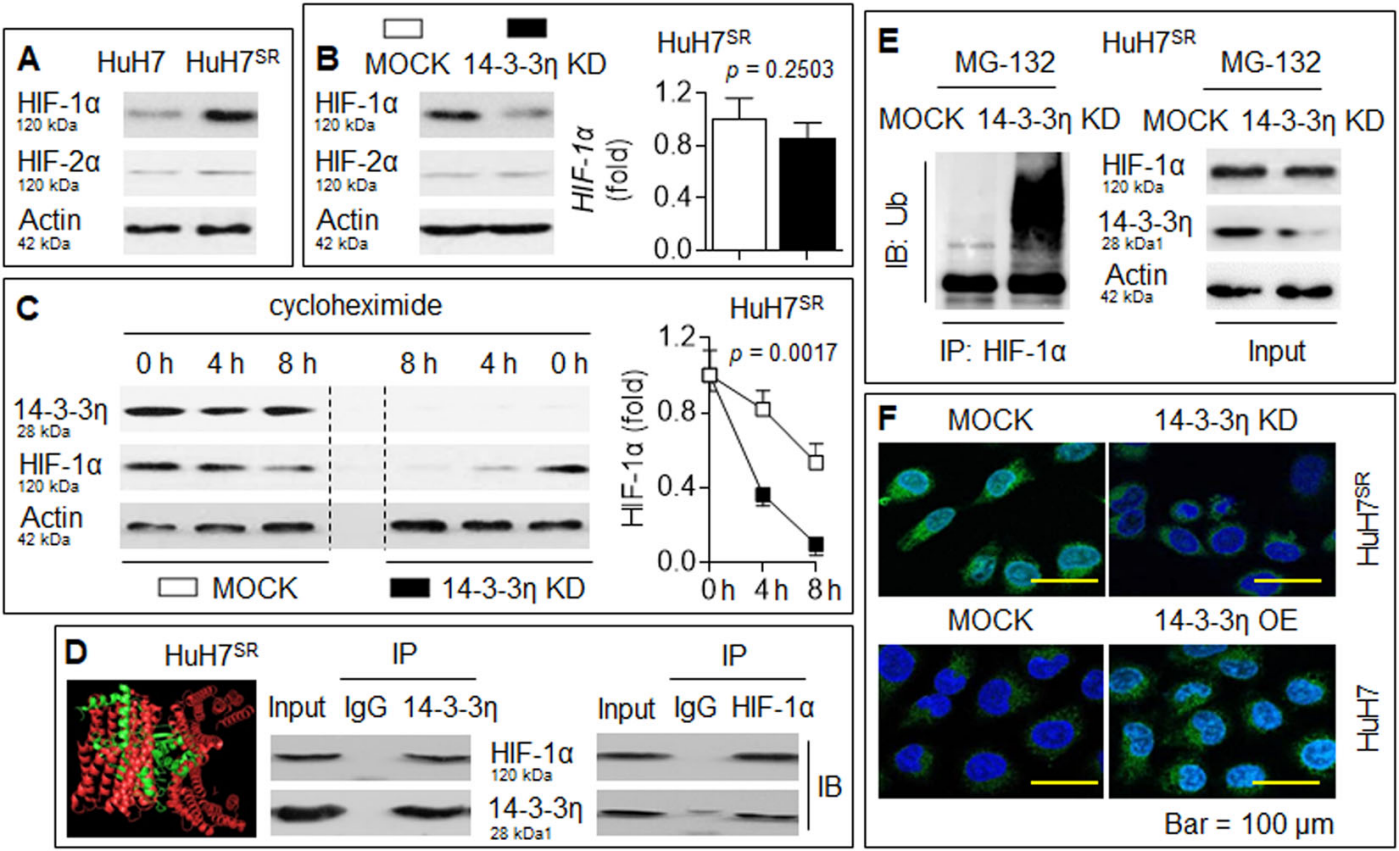
14-3-3η stabilized and activated HIF-1α. **a** IB analysis of the expressions of HIF-1α and HIF-2α in HuH7 and HuH7^SR^ cells. **b** HuH7^SR^ cells were transfected by scrambled or 14-3-3η siRNA, IB (left) and qRT-PCT (right) analysis of the expressions of HIF-1α or HIF-2α. **c** After HuH7^SR^ cells were transfected by scrambled or 14-3-3η siRNA, they were treated by cycloheximide for 0, 4, or 8 h. IB analysis of the expression of HIF-1α. **d** Computer-docking (left) and IP analysis (right) of the relationship between 14-3-3η and HIF-1α proteins in HuH7^SR^ cells. **e** After HuH7^SR^ cells were transfected by scrambled or 14-3-3η siRNA, they were treated by MG-132 for 2 h, IP analysis of the ubiquitination of HIF-1α. **f** HuH7^SR^ cells were transfected by scrambled or 14-3-3η siRNA, while HuH7 cells were transfected by scrambled or 14-3-3η plasmid. Immunostaining analysis of the expression and intracellular distribution of HIF-1α

In HCC, sorafenib aggravates microenvironmental hypoxia while exerting anti-tumor effects and then induces the enhancement of CSC properties by activating HIF-1α, which leads to drug resistance^5,6^. Here, we found that the expression of HIF-1α, but not HIF-2α, was increased in HuH7^SR^ cells compared with their parental counterparts (Fig. 2a). The knockdown of 14-3-3η in HuH7^SR^ cells significantly reduced the protein level of HIF-1α, but its mRNA level remained stable (Fig. 2b). We then used cycloheximide to stop protein synthesis and examined the turnover of HIF-1α. As shown in Fig. 2c, HIF-1α was degraded at a much faster rate in 14-3-3η siRNA-transfected HuH7^SR^ cells. Therefore, we hypothesized that 14-3-3η regulates HIF-1α at the posttranscriptional level.

Using computer docking software (PyMOL), we found that 14-3-3η could bind to HIF-1α, and this binding was further confirmed via an IP assay (Fig. 2d). We then treated scrambled- or 14-3-3η siRNA-transfected HuH7^SR^ cells with the proteasome inhibitor MG-132 to stop proteasomal protein degradation. As shown in Fig. 2e, the knockdown of 14-3-3η enhanced the ubiquitination level of HIF-1α. Moreover, an immunostaining assay showed that the knockdown of 14-3-3η in HuH7^SR^ cells decreased the cytosol/nuclear fluorescence intensity of HIF-1α, whereas the overexpression of 14-3-3η in the parental HuH7 cells increased the expression/nuclear translocation of HIF-1α (Fig. 2f). These findings collectively suggested that 14-3-3η activated HIF-1α at the posttranscriptional level via binding and thereby inhibited ubiquitin-dependent proteasome protein degradation in HCC cells.

### miR-16 repressed HIF-1α via a targeted intervention of 14-3-3η

**Fig. 3:**
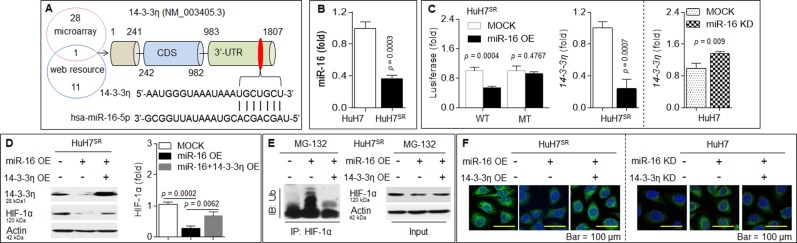
miR-16 repressed HIF-1α via a targeted intervention of 14-3-3η. **a** Potential miRNAs of *14-3-3η* as predicted by TargetScan, and the target sequences of miR-16 in the 3′-UTR of *14-3-3η* mRNA. **b** qPCR analysis in triplicate of the expression of miR-16 in HuH7 and HuH7^SR^ cells. **c** (Left) HuH7^SR^ cells were co-transfected by scrambled or miR-16 mimic in the presence of pGL3-14-3-3η 3′-UTR (WT or MT)-Luc constructs. Luciferase reporter assay analysis in triplicate of the effects of miR-16 on *14-3-3η*-3′-UTR. HuH7^SR^ cells were transfected by scrambled or miR-16 mimic (middle), while HuH7 cells were transfected by scrambled or anti-miR-16 (right), after then, the expression of *14-3-3η* mRNA was determined in triplicate via qPCR. **d**–**f** HuH7^SR^ cells were transfected by scrambled, miR-16 mimic, or miR-16 mimic plus 14-3-3η plasmid. **d** IB analysis of the expressions of 14-3-3η and HIF-1α with different treatments. **e** IP analysis of the ubiquitination of HIF-1α. **f** Immunostaining analysis of the expression and intracellular distribution of HIF-1α

As mentioned above, a combined miRNA microarray analysis using web-based miRNA resources predicted that miR-16 can bind to the 3′-UTR of 14-3-3η mRNA (Fig. 3a). Interestingly, the expression of miR-16 in HuH7^SR^ was significantly lower than that in parental HuH7 cells (Fig. 3b). Therefore, we first constructed pGL3-*14-3-3η*-3′-UTR (WT or MT)-Luc constructs and found that the cotransfection of the miR-16 mimic with WT but not MT constructs led to a significant decrease in luciferase activity (Fig. 3c). We subsequently determined the functional association between the binding effects of miR-16s and the expression of *14-3-3η* mRNA. As shown in Fig. 3c, the overexpression of miR-16 in HuH7^SR^ cells decreased the expression of *14-3-3η*, whereas the inhibition of miR-16 in HuH7 cells increased *14-3-3η* expression. Furthermore, miR-16 overexpression in HuH7^SR^ cells enhanced the ubiquitination level but decreased the expression/activation of HIF-1α, whereas the recovery of 14-3-3η attenuated these effects (Fig. 3d–f). In parental HuH7 cells, the inhibition of miR-16 increased the expression/activation of HIF-1α, and these effects were attenuated by 14-3-3η knockdown (Fig. 3f). Collectively, these results suggested that decreased expression of miR-16 abolished the targeted intervention of 14-3-3η and thereby activated HIF-1α.

### miR-16/14-3-3η regulated CSC properties and sorafenib resistance

**Fig. 4:**
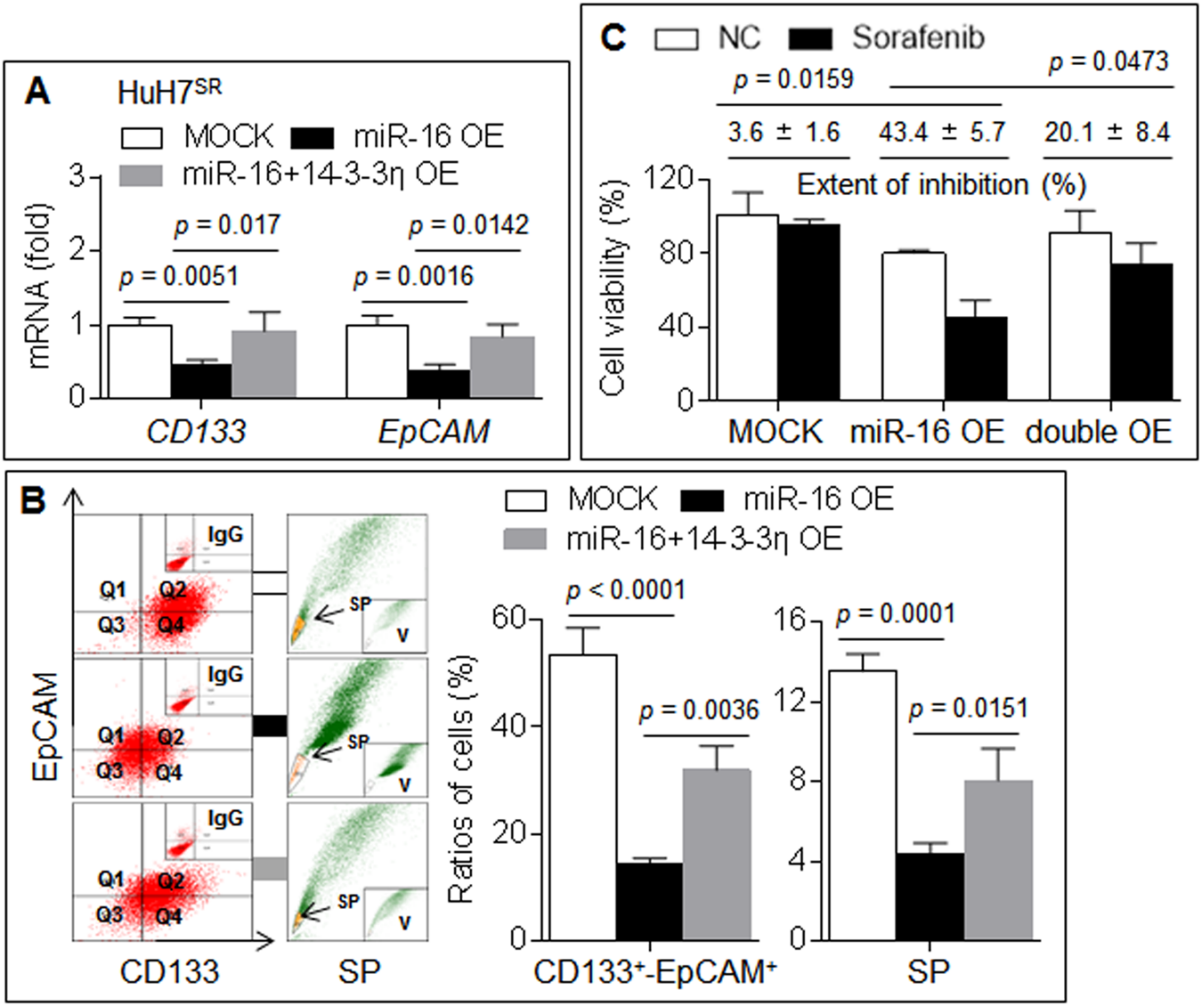
miR-16/14-3-3η regulated CSCs properties and sorafenib resistance. **a**, **b** HuH7^SR^ cells were transfected by scrambled, miR-16 mimic, or miR-16 mimic plus 14-3-3η plasmid. **a** qRT-PCR analysis of the expressions of *CD133* and *EpCAM* mRNAs. **b** Flow cytometry analysis in triplicate of the ratio of CD133^+^-EpCAM^+^ and SP cells. **c** After HuH7^SR^ cells were pre-transfected as described above, they were treated by sorafenib. Cell viabilities were analyzed in triplicate by CCK-8 solution

We investigated the effects of miR-16/14-3-3η on CSC properties. Our analysis showed that forced expression of miR-16 in HuH7^SR^ cells decreased the expression of CD133 and EpCAM and reduced the ratios of CD133^+^–EpCAM^+^ and SP cells and that the recovery of 14-3-3η inhibited these effects (Fig. 4a, b). We subsequently confirmed that the recovery of 14-3-3η partially reversed the miR-16-induced response to sorafenib in HuH7^SR^ cells (Fig. 4c). Based on the abovementioned results, our in vitro study indicated that the targeted intervention of 14-3-3η by miR-16 induced the ubiquitin-dependent degradation of HIF-1α, which led to the attenuation of CSC properties and the reversal of sorafenib resistance.

### Confirmation of the in vitro data in a xenograft model

**Fig. 5:**
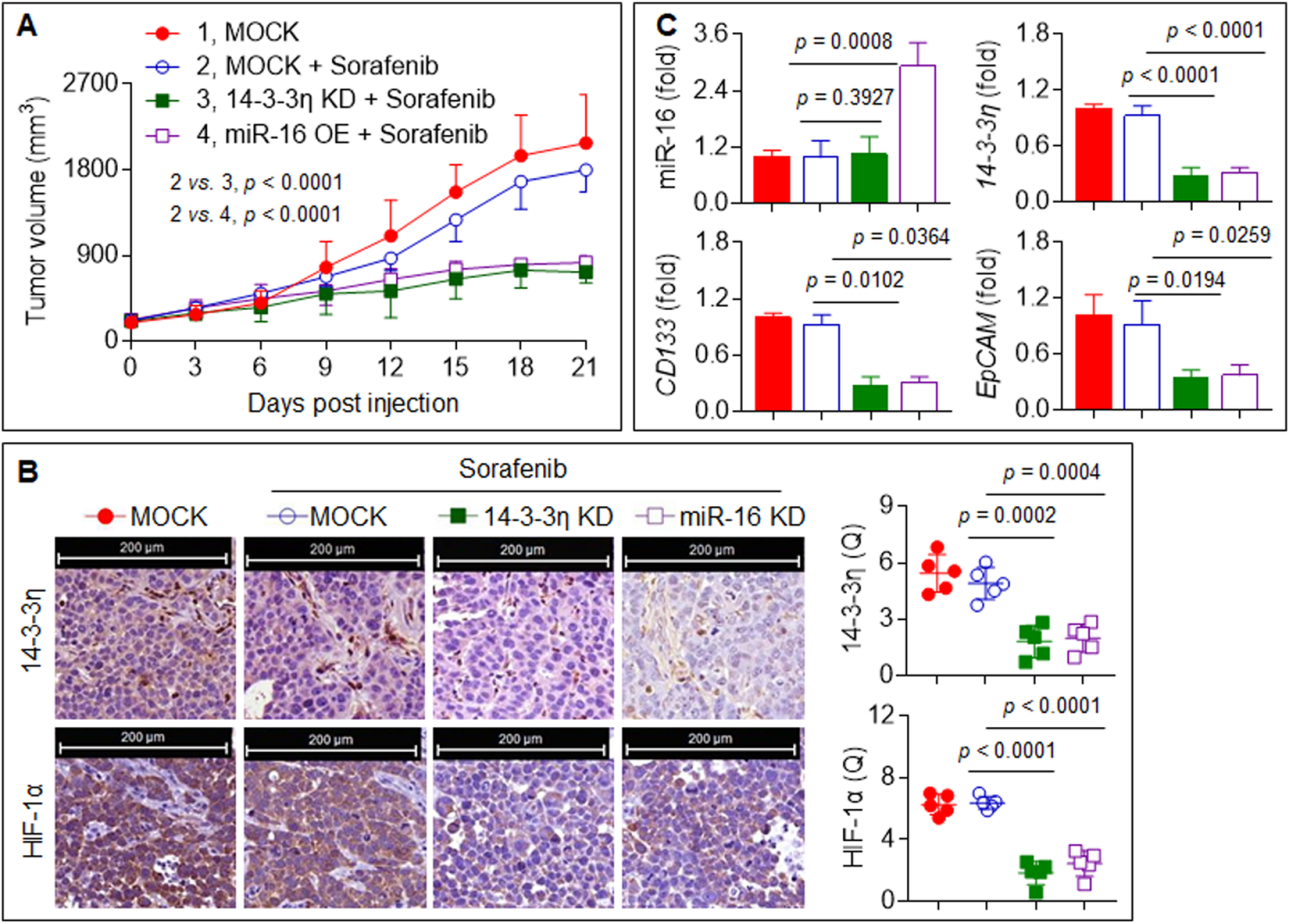
Confirmation the in vitro data in a xenograft model. The HuH7^SR^ cells xenograft tumors were treated by sorafenib alone, sorafenib plus miR-16 agomir, or sorafenib plus 14-3-3η siRNA. **a** The volumes of xenografts tumors in different treatments described above. **b** IHC staining of the 14-3-3η and HIF-1α (Note: each point represented the mean of one xenografts tumor section calculating in 5 high-power fields). **c** qRT-PCR analysis of the expressions of miR-16, *14-3-3η*, *CD133*, and *EpCAM* mRNAs in xenografts tumors

As shown in Fig. 5a, the treatment of the xenografts with sorafenib alone mildly inhibited tumor growth. However, sorafenib treatment combined with 14-3-3η knockdown or miR-16 overexpression facilitated the sorafenib-induced inhibition of tumor growth. Moreover, sorafenib plus 14-3-3η siRNA or sorafenib plus miR-16 agomir significantly decreased the expression of 14-3-3η, HIF-1α, CD133, and EpCAM compared with sorafenib treatment alone (Fig. 5b, c).

### Clinical significance of miR-16 and 14-3-3η in HCC

**Fig. 6:**
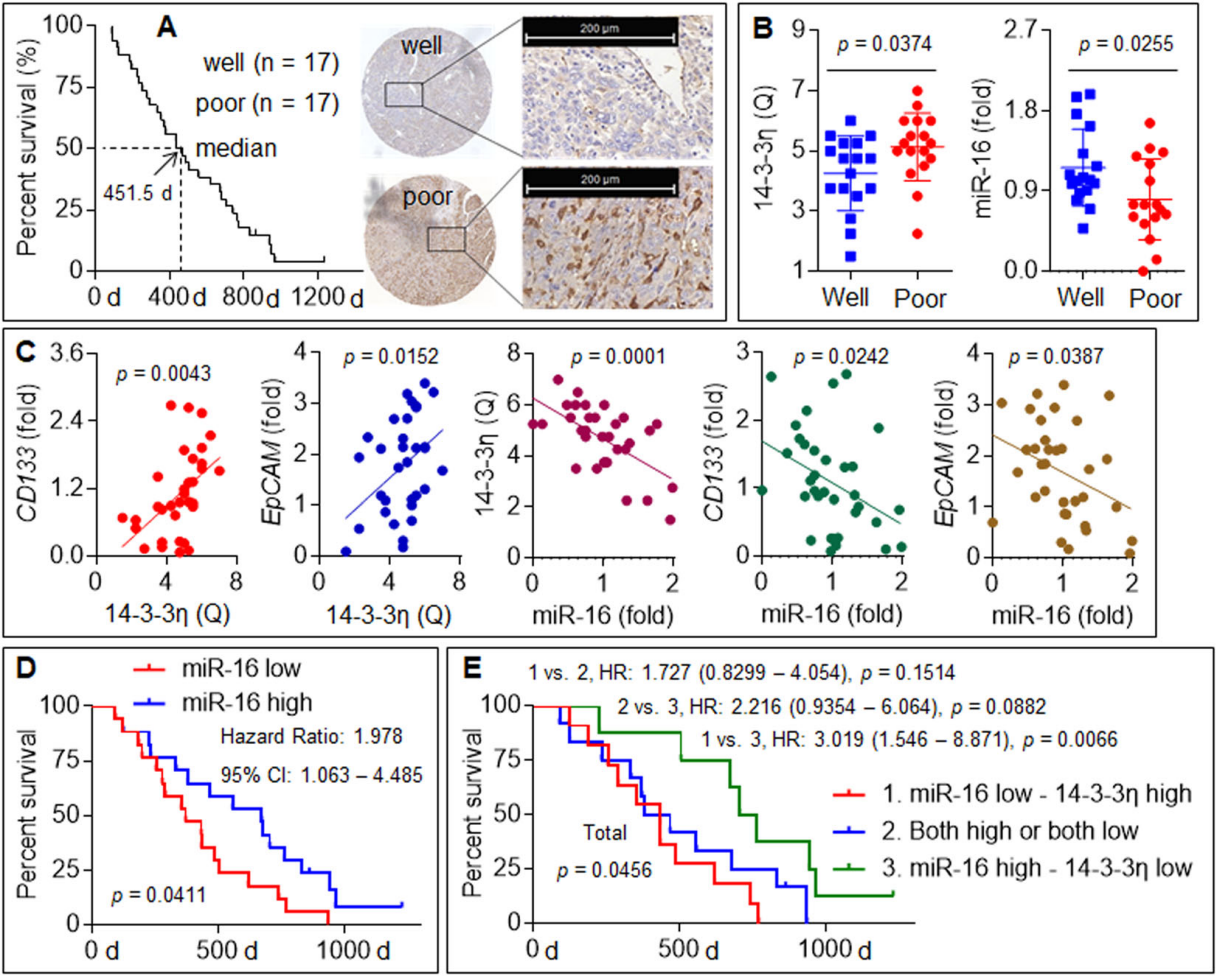
Clinical significance of miR-16 and 14-3-3η in HCC. **a** IHC staining (right) of 14-3-3η in HCC tissues with different differentiated prognosis based on the median survival (left). **b** qRT-PCR analysis of 14-3-3η and miR-16 in HCC tissues with different differentiated prognosis based on the median survival. **c** Analysis of the correlations between miR-16 and 14-3-3η, CD133, or EpCAM. **d**, **e** Kaplan–Meier analysis of the prognostic significances of 14-3-3η and miR-16

In 34 patients with advanced recurrent HCC receiving combined sorafenib therapy, the expression of 14-3-3η in patients with a poor prognosis was significantly higher compared with that in patients with a good prognosis, whereas the expression of miR-16 showed the opposite phenomenon (Fig. 6a, b). Moreover, significant positive correlations were found between the expression of 14-3-3η and that of CD133 or EpCAM; in contrast, markedly negative correlations were found between the expression of miR-16 and that of 14-3-3η, CD133, or EpCAM (Fig. 6c). Finally, the cohort of 34 patients with advanced recurrent HCC were divided into “high miR-16 expression/low 14-3-3η expression”, “high miR-16 expression/high 14-3-3η expression or low miR-16 expression/low 14-3-3η expression”, and “low miR-16 expression/high 14-3-3η expression” groups. A Kaplan–Meier survival analysis also showed that the patients in the “low miR-16 expression/high 14-3-3η expression” group exhibited worse survival than those in the “high miR-16 expression/low 14-3-3η expression” group (Fig. 6d, e). Collectively, these results indicated that the silencing of miR-16 in HCC patients might contribute to the upregulation of 14-3-3η and thereby lead to resistance to sorafenib therapy.

## Discussion

Sorafenib is currently regarded as the only effective chemotherapy regimen for advanced HCC^27^, but the overall survival after this treatment remains limited due to the frequent development of resistance to sorafenib^28^. In general, sorafenib resistance includes HCC cell resistance and microenvironmental resistance. Analyses of the underlying molecular mechanisms have revealed that abnormal phosphorylation modifications (EGFR, ERK, AKT, and STAT-3), the epithelial-to-mesenchymal transition (EMT), CSC properties, and angiogenesis enhancement are involved in the resistance of HCC cells to sorafenib, while the heterogeneity of tumor vessels (lack of VEFGR1/2) and the advancement of hepatic fibrosis, as well as inflammation and hypoxia, contribute to microenvironmental or vascular resistance^29–31^.

miR-16 and miR-15 are highly conserved miRNAs in the miR-15 family and are found at high levels in normal tissues. Several binding sites, including c-Myc, c-Myb, and PPAR, function in coordination with miR-15/16 to regulate various biological processes^32^. The downregulation of miR-16 reportedly results in escape from cellular apoptosis, which might exert an influence on tumorigenesis and tumor progression^33^. Furthermore, previous studies have demonstrated that miR-16 serves as a tumor suppressor and that a lack of miR-16 might render tumors resistant to chemotherapy drugs such as fluorouracil and cisplatin^25,26^. Furthermore, cells can regain sensitivity to anti-tumor drugs with high miR-16 expression in gastric carcinoma, lung carcinoma, and breast cancer^25,34^, but the correlation between miR-16 and sorafenib resistance remains unclear. We hypothesize that miR-16 is a competent miRNA that reverses sorafenib resistance by targeting the 3′-UTR of 14-3-3η and thereby inhibits 14-3-3η/HIF-1α/CSC properties.

The 14-3-3 protein family has been described as a family of scaffolding proteins that participate in many signaling pathways. Specifically, 14-3-3 proteins act as enzymes that regulate EGFR signaling and are colocalized with EGFR along the plasma membrane^35^. The upregulation of 14-3-3ζ activates PI3K, and thus, Akt signaling can be facilitated^36,37^. Moreover, 14-3-3 proteins can bind to many downstream proteins in the PI3K/Akt pathway, such as Bad and β-catenin. In addition, 14-3-3 proteins can promote MAPK signaling and are important for the maintenance of activation through the modification of phosphorylation^38,39^. Furthermore, 14-3-3 proteins have been implicated in the intracellular distribution of client proteins^40,41^. In fact, the 14-3-3ζ protein can interact with β-catenin and promote its translocation from the cytosol to the nucleus^42^ and is also involved in the nuclear exclusion of FoxO3 when binding to its phosphorylated form^43^. The 14-3-3σ protein can bind to COP1, and this binding is required for its translocation to the cytoplasm^44^. Due to the complex interaction between 14-3-3 proteins and signaling networks, a series of cellular functions are altered in response to internal and external stimulation. A positive correlation between 14-3-3ζ and HIF-1α has been demonstrated and might play a role in HCC progression and metastasis^36,45^. We found that 14-3-3η regulated the stabilization and nuclear translocation of HIF-1α in HCC cells.

Hypoxia is a characteristic of solid tumors and an important stem cell niche, particularly in HCC^46^. The von Hippel–Lindau (VHL) E3 ubiquitin ligase plays a classic role in the regulation of HIF-1α under normoxic conditions, but the repressive effect is attenuated by the inhibition of proline hydroxylation under hypoxia^5,47^. A previous study revealed that the background expression level of VHL in HuH7 cells is very low^6^. Here, we hardly detected the expression of this protein in both HuH7 and HuH7^SR^ cells, and the knockdown of 14-3-3η in HuH7^SR^ cells slightly increased VHL expression (data not shown). Based on these findings, we hypothesized that VHL participates in the 14-3-3η-regulated stabilization of HIF-1α and that a VHL-independent mechanism might also be involved in this process. A recent study revealed that parkin is a novel E3 ligase for HIF-1α (the in-between-RING domain is required for the interaction) that ubiquitinates HIF-1α in a VHL-independent manner^48^. Based on the computer docking results obtained in the present study, the combination of parkin and HIF-1α can be blocked by 14-3-3η, and based on the results from a previous study that showed that 14-3-3η is a specific inhibitor of parkin^49^, we hypothesized that blockage of the parkin-mediated ubiquitin-dependent proteasome protein degradation pathway is also involved in the 14-3-3η-mediated stabilization of HIF-1α.

Our present study revealed that 14-3-3η maintained the CSC properties of HuH7^SR^ cells. Interestingly, these cells exhibited a mesenchymal-like morphology (data not shown). Based on our previous findings that EMT cells acquire stem cell-like traits and that CSCs exhibit a mesenchymal-like appearance^21^, we hypothesized that the induction of mesenchymal-like changes is involved in the 14-3-3η-mediated maintenance of sorafenib resistance. Importantly, we previously revealed that 14-3-3η is a novel characteristic growth-promoting factor in HCC, including both tumors and intratumoral vessels^16^. Increasing evidence indicates that HCC-derived endothelial cells exhibit a mesenchymal-like morphology, enhanced angiogenic activity, and resistance to sorafenib^50^. Therefore, further studies should be conducted with an emphasis on the functions of 14-3-3η in vascular resistance and with a particular focus on the involvement of the endothelial-to-mesenchymal transition.

## Conclusion

**Fig. 7:**
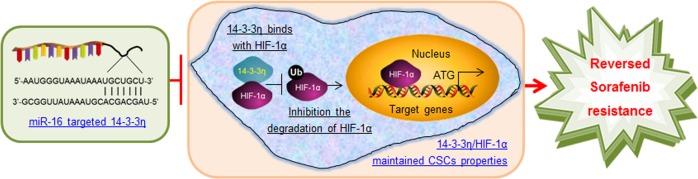
Reversal of sorafenib resistance in HCC: epigenetically-regulated disruption of 14-3-3η/HIF-1α

In summary, this work demonstrates that the silencing of miR-16 or an increase in the 14-3-3η level constitutes one of the main mechanisms underlying the upregulation of HIF-1α/CSC properties and subsequently the induction of sorafenib resistance (Fig. 7) and that miR-16 and 14-3-3η could be potential therapeutic targets and biomarkers for predicting the response to sorafenib treatment.

## Materials and methods

### Ethics approval and consent to participate

This study was approved by the Medical Ethics Committee of Nanjing Medical University, and the written informed consent was obtained from each patient^19^. All in vivo protocols were approved by the Nanjing Medical University Institutional Animal Care and Use Committee (2017-KY010).

### Cell lines and construction of sorafenib-resistant cells

The HuH7 cell line was obtained from Institute of Biochemistry and Cell Biology (Shanghai, China). Cells were maintained in a 37 °C humidified incubator with 5% CO_2_ in Dulbecco’s Modified Eagle’s Medium (Gibco, NY, USA), supplemented with 10% fetal bovine serum (FBS), 100 U/ml penicillin, 100 μg/ml streptomycin (Gibco)^51^. For the generation of a sorafenib-resistant line, as described previously^4^, HuH7 cells were treated with 10 μM sorafenib for 72 h, and viable cells remaining attached to the dish were harvested and sub-cultured. This process was continued for five rounds. Resistant cells were maintained in the continuous presence of 10 μM sorafenib.

### Patients and xenograft in nude mice

A cohort of 34 patients with advanced recurrent HCC receiving combined sorafenib treatment and transarterial chemoembolization therapy were analyzed (the clinic-pathologic data were as described previously^16^). For the animal model, BALB/c nude mice were obtained from the SLRC Laboratory Animal Center (Shanghai, China) and kept in a specific pathogen-free and temperature-controlled environment (20–22 °C) with a 12 h light–dark cycle and free access to drinking water and chow. For the xenograft study, 2 × 10^6^ cells in 100 μl matrigel were injected subcutaneously into the right armpit of mice for 3 weeks as described previously^16^. We used 60 mg/kg BW of sorafenib (Selleckchem, Houston, TX, USA) via gavage, with scrambled, 14-3-3η siRNA (Santa Cruz Biotechnology, CA, USA), or miR-16 agomir (RiboBio, Guangdong, China) via intratumoral injection every 3 days^4,52^. Tumor volumes were calculated using the formula: *V* = 1/2 (width^2^ × length). After 21 days, the mice were killed, and tumor tissues were removed for further investigation.

### Transfection and luciferase reporter assay

Commercial scrambled, 14-3-3η siRNA, miR-16 mimic, and anti-miR-16 are listed in Supplementary Table S1. The pcDNA-3.1-14-3-3η-FLAG plasmid that overexpressed both 14-3-3η and FLAG was created by inserting the coding sequences of 14-3-3η (YWHAH, 741 bp) into pcDNA3.1 plasmid, followed by adding a FLAG-tag at its N-terminal (Generay Biotech Co. Ltd., Shanghai, China). Cells were seeded in 6-well plates at a density of 1 × 10^5^ per well, followed by transient transfection using the Lipofectamine 2000 reagent (Invitrogen, Carlsbad, CA, USA), according to the manufacturer’s protocol. After transfection, cells were cultured in fresh medium supplemented with 10% FBS for another 24 h before being used for other experiments. For the luciferase reporter assay, the pGL3-14-3-3η 3′-UTR (wild type, WT; or mutant, MT)-Luc constructs were synthesized by Shuntian Bio Co. (Shanghai, China). The plasmid phRL-tk containing the Renilla luciferase gene was purchased from Promega (Madison, WI, USA). As we described previously^18,20^, after cells were plated in 24-well culture dishes for 48 h, they were co-transfected using Luc constructs plus miR-16 mimic. Cells were then lysed with passive lysis buffer, and the lysates were analyzed immediately using a 96-well plate luminometer (Berthold Detection System, Pforzheim, Germany)^53^.

### Determination of cell viability

A total of 2 × 10^3^ cells was seeded in 96-well plates for 24 h, and then treated as indicated in “Results”. The cells were then incubated with 20.0 μl of CCK-8 solution (Dojindo Molecular Technologies, Inc., Kumamoto, Japan) for another 4 h. The absorbance at 450 nm was measured with a multi-well plate reader (Model 680, Bio-Rad, USA)^16^. Cell viability and inhibition are calculated using the data from measured absorbance.

### Flow cytometry determining CSCs properties

To determine the side population (SP) ratio, treated cells were resuspended in DMEM/F-12 medium (Gibco) containing 2% FBS, and stained with 5 μg/ml Hoechst 33342 (Sigma, St. Louis, MO, USA) in the presence or absence of 50 μM verapamil (Sigma) at 37 °C for 90 min, followed by counterstaining with 2 μg/ml PI. To determine the CD133^+^–EpCAM^+^ ratio, treated cells were incubated at 4 °C in the dark for 40 min with fluorescence-conjugated monoclonal antibodies obtained from BD Biosciences against human CD133-FITC and EpCAM-Percp-Cy5.5, and their isotype IgG1. Experiments were performed using a FACS-Aria III system (BD), and analyzed via a Flow-Jo software (Ashland, OR, USA)^54^.

### Quantitative real-time polymerase chain reaction (qRT-PCR)

Primers used are listed in Supplementary Table S2. Total RNA was isolated using Trizol (Invitrogen), followed by transcription into cDNA using AMV reverse transcriptase (Promega). The PCR was performed using the Applied Biosystems 7300HT machine (Applied Biosystems, CA, USA) and MaximaTM SYBR Green/ROX qPCR Master Mix (Fermentas, MA, USA). The fold change in expression of each gene was calculated using the comparative threshold cycle (Ct) method with the formula 2^−(ΔΔCt)^ ^16^.

### Immunostaining

Cells were fixed in 10% formalin solution (4% paraformaldehyde; Beyotime Co. Ltd.) for 5 min followed by permeabilization in PBS containing 0.1% Triton X-100 for another 5 min. The cells were then washed three times with PBS, blocked with 5% non-fat milk in PBS for 30 min, and incubated with rabbit-anti-HIF-1α (1: 200) antibody at 4 °C overnight. Following incubation, cells were washed three times with PBS and incubated for 1 h in the presence of FITC-conjugated goat-anti-rabbit (green) secondary antibody (Beyotime Co. Ltd.; dilutions, 1:500). After washing with PBS, the nuclei were stained by adding 4′,6-diamidino-2-phenylindole (DAPI, Beyotime) for 10 min. Cells were observed and pictured under a Zeiss-700B laser scanning confocal microscope (Zeiss Co. Ltd., Oberkochen, Germany).

### Immunoblotting (IB) and immunoprecipitation (IP)

Total protein was extracted by RIPA buffer (Beyotime), and protein concentrations were measured using the BCA kit (Beyotime). Afterwards, proteins (20 μg) were separated using 10% sodium dodecyl sulfate-polyacrylamide gel electrophoresis followed by transfer to polyvinylidene fluoride membranes (Millipore, Billerica, USA). After blocking, membranes were incubated with the primary antibody (Supplementary Table S3) at 4 °C overnight, and incubated with horseradish peroxidase-conjugated secondary antibodies (Beyotime) for 1 h. The immune complexes were detected using enhanced chemiluminescence (Cell Signaling Technology, MA, USA). For IP, after proteins were incubated with primary antibody at 4 °C overnight, they were incubated with IgG Sepharose beads (Beyotime) at 4 °C for another 12 h. After then, the supernatants were removed and the beads were washed, resuspended, and analyzed using an IB assay^16^.

### Immunohistochemistry (IHC)

Sections mounted on silanized slides were dewaxed in xylene; dehydrated in ethanol; boiled in 0.01 M citrate buffer (pH 6.0) for 20 min in a microwave oven; and then incubated with 3% hydrogen peroxide for 5 min. After washing with PBS, sections were incubated in 10% normal bovine serum albumin for 5 min, followed by incubation with primary antibody at 4 °C overnight. The slides were then incubated with a horseradish peroxidase-conjugated secondary antibody at room temperature for another 30 min. Samples were then visualized using diaminobenzadine, dehydrated, cleared, mounted, and photographed under a panoramic-scan digital slice scanning system (3DHISTECH Co. Ltd., Budapest, Hungary). The graphs were analyzed using Image-Pro-Plus 6.0 software. Quantitation was performed by two independent researchers who were blinded regarding patient details. The immunostaining score was semi-quantified using Quick-score (*Q*-score) based on intensity and heterogeneity as described previously^16^.

### Statistical analysis

Data are presented as the mean ± SD, and compared using a GraphPad 6.0 software (San Diego, CA, USA). The differences were analyzed using Student’s *t* test, one-way analysis of variance followed by Dunnett’s *t* test, or two-way analysis of variance followed by Sidak’s multiple comparisons test. Survival curves were estimated using the Kaplan–Meier method, and evaluated by the log-rank test. The *p* values <0.05 were considered statistically significant.

## Supplementary information

Table S1-S3

Author contributions
