# Supplementary material for: Reversal of sorafenib resistance in hepatocellular carcinoma: epigenetically regulated disruption of 14-3-3η/hypoxia-inducible factor-1α
Source: Cell Death Discov. 2019 Jul 19;5:120. doi: 10.1038/s41420-019-0200-8 (PMC6642098; doi:10.1038/s41420-019-0200-8)
Supplement: Supplementary file 1 — Table S1-S3 [file 41420_2019_200_MOESM1_ESM.docx]

**Table. S1. siRNAs used in this study**

| siRNAs | web link | source | used |
| --- | --- | --- | --- |
| 14-3-3η | https://datasheets.scbt.com/sc-43581.pdf | Santa Cruz  Biotechnology | 20 nM (*in vitro*)  100 nM (*in vivo*) |

**Note:** The mimic [(http://www.ribobio.com/sitecn/product_info.aspx?id=208317](http://www.ribobio.com/sitecn/product_info.aspx?id=208317))) and agomir [(http://www.ribobio.com/sitecn/product_info.aspx?id=219625](http://www.ribobio.com/sitecn/product_info.aspx?id=219625))) for miR-16 were designed and synthesized by RiboBio Co. The concentration of miR-16-mimic used in the in vitro study was 20 nM, while the concentration of miR-16-agomir used in the in vivo study was 60 nM.

**Table. S2. Primers used in this study**

| mRNAs | primers (5’-3’) | mRNAs | primers (5’-3’) |
| --- | --- | --- | --- |
| *14-3-3η* | CCTGCCTCTTAGCCAAAC (F) CTCCTGCTTCTTCATCCTG (R) | *EpCAM* | AAGGAGAAACAGGAAACCTC (F) ACAGACACAGTCCAACTTCC (R) |
| *CD133* | TCGGAAACTGGCAGATAGC (F)  GAACGCCTTGTCCTTGGT (R) | *HIF-1α* | GCCGCCCGCCGTGAAGAC (F)  GAAGTGGCAACTGATGAGCA (R) |
| *HIF-2α* | CACCAAGGGTCAGGTAGTAAG GGTTGCGAGGGTTGTAGAT |  |  |

**Table. S3. Antibodies used in this study**

| antibodies | web link | source | used |
| --- | --- | --- | --- |
| 14-3-3η | https:/[/www.cellsignal.com/products/primary-ant](http://www.cellsignal.com/products/primary-ant) ibodies/14-3-3-h-antibody/9640?_=15234554246  11&Ntt=14-3-3&tahead=true | Cell Signaling  Technology | 1: 1000 (IB)  1: 50 (IHC) |
| HIF-1α | https:/[/www.cellsignal.com/products/primary-ant](http://www.cellsignal.com/products/primary-ant) ibodies/hif-1a-d1s7w-xp-rabbit-mab/36169?_=15  23455437257&Ntt=HIF-1&tahead | Cell Signaling  Technology | 1: 1000 (IB) |
| HIF-2α | https:/[/www.cellsignal.com/products/primary-ant](http://www.cellsignal.com/products/primary-ant) ibodies/hif-2a-d6t8v-rabbit-mab/59973?site-searc  h-type=Products | Cell Signaling  Technology | 1: 1000 (IB) |
| Ubiquitin | https:/[/www.novusbio.com/products/ubiquitin-an](http://www.novusbio.com/products/ubiquitin-an)  tibody-ubi-1_nb300-130 | Novus | 1: 1000 (IB) |
| β-Actin | <http://www.beyotime.com/product/AA128.htm> | Beyotime | 1: 1000 (IB) |
