# Supplementary material for: Reversal of sorafenib resistance in hepatocellular carcinoma: epigenetically regulated disruption of 14-3-3η/hypoxia-inducible factor-1α
Source: Cell Death Discov. 2019 Jul 19;5:120. doi: 10.1038/s41420-019-0200-8 (PMC6642098; doi:10.1038/s41420-019-0200-8)
Supplement: Supplementary file 2 — Author contributions [file 41420_2019_200_MOESM2_ESM.docx]

**Author contributions**

Y.L. , L.L. , J.Z. , and L.Y. conceived and designed the study. Y.Q. , W.S. , Y.X. , and Q.L. performed the *in vitro* experiments (Figs. 1 to 4). Y.D. , R.J. , H.Y. , and L.J. performed the *in vivo* experiments (Fig. 5). Y.Y. , M.J. , and G.H. collected and analysed the HCC samples (Fig. 6). Y.Q. , Y.Y. , M.J. , W.S. , and Y.D. analyzed the data and drafted the manuscript. J.Z. , L.Y. , L.L. , and Y.L. revised the manuscript.
